# Effects of PUFA-Rich Dietary Strategies on Ruminants’ Mammary Gland Gene Network: A Nutrigenomics Review

**DOI:** 10.3390/metabo13010044

**Published:** 2022-12-27

**Authors:** Panagiota Kyriakaki, Foivos Zisis, Athanasios C. Pappas, Alexandros Mavrommatis, Eleni Tsiplakou

**Affiliations:** Laboratory of Nutritional Physiology and Feeding, Department of Animal Science, School of Animal Biosciences, Agricultural University of Athens, Iera Odos 75, 11855 Athens, Greece

**Keywords:** antioxidant, immune system, gene networks, fat synthesis, marine lipids, milk, plant-derived lipids, PUFAs

## Abstract

Although the inclusion of polyunsaturated fatty acids (PUFAs) in ruminants’ diets appears to be a well-documented strategy to enrich milk with PUFAs, several gene networks that regulate milk synthesis and mammary gland homeostasis could be impaired. The objective of this literature review is to assess the effects of nutritional strategies focused on enriching milk with PUFAs on gene networks regulating mammary gland function and lipogenesis, as well as the impact of feed additives and bioactive compounds with prominent antioxidant potential on immune-oxidative transcriptional profiling, as a part of mammary gland homeostasis and health. The findings support the conclusion that PUFAs’ inclusion in ruminants’ diets more strongly downregulate the stearoyl-CoA desaturase (*SCD*) gene compared to other key genes involved in de novo fatty acid synthesis in the mammary gland. Additionally, it was revealed that seed oils rich in linoleic and linolenic acids have no such strong impact on networks that regulate lipogenic homeostasis compared to marine oils rich in eicosapentaenoic and docosahexaenoic acids. Furthermore, ample evidence supports that cows and sheep are more prone to the suppression of lipogenesis pathways compared to goats under the impact of dietary marine PUFAs. On the other hand, the inclusion of feed additives and bioactive compounds with prominent antioxidant potential in ruminants’ diets can strengthen mammary gland immune-oxidative status. Considering that PUFA’s high propensity to oxidation can induce a cascade of pro-oxidant incidences, the simultaneous supplementation of antioxidant compounds and especially polyphenols may alleviate any side effects caused by PUFA overload in the mammary gland. In conclusion, future studies should deeply investigate the effects of PUFAs on mammary gland gene networks in an effort to holistically understand their impact on both milk fat depression syndrome and homeostatic disturbance.

## 1. Introduction

The fast pace of everyday life has rapidly shifted nutritional habits to foods with a high content of saturated fatty acids (SFAs) or sugars, thus increasing the risk for cardiovascular diseases [1], diabetes [2], and obesity [3]. Conversely, the influence of nutrition on health is summarized by Hippocrates’ quote “leave the drugs on the chemists’ pot, if you can heal a patient with food” [4]. In other words, the established idea that nutrition should just nourish people needs to be substituted with a more holistic approach that is not just focused on covering needs. Nowadays, nutraceuticals and functional foods with a high content of bioactive compounds, such as omega-3 polyunsaturated fatty acids (PUFAs) and antioxidants, have received considerable attention from both consumers and the industry. 

In most developed countries, milk and dairy products are the major source of SFAs in the human diet. For this reason, there has been considerable interest in decreasing the proportions of SFAs in milk fat as a means of meeting national targets without requiring substantive changes in consumers’ eating habits. Several reviews have summarized the potential of dietary strategies on modifying the milk fat composition of lactating ruminants [5,6,7]. Amongst these strategies, dietary supplementation with unconventional feedstuffs rich in unsaturated fatty acids (UFA), such as soybean oil, linseed oil, sunflower oil, safflower oil, rapeseed, canola seeds, fish oil, and microalgae, have been well-documented in dairy ruminants, and their impact on milk fortification with PUFAs is now clear. However, scarce information exists about their impact on several other metabolic and cellular functions related to milk synthesis and the organisms’ homeostasis. In this context, a meta-transcriptomic analysis of the bovine mammary gland unveiled that more than 1000 genes are differentially expressed when Holstein cows are fed 5% linseed, and it argued that PUFA-rich diets downregulated genes involved in fatty acid/lipid synthesis and lipid metabolism pathways [8]. On the other hand, high levels of PUFAs in ruminants’ diet can also cause some detrimental effects, such as oxidative stress, and consequently, homeostatic imbalances in the mammary gland due to the high propensity of PUFAs to nonenzymatic and enzymatic oxidation [9]. Considering the aforementioned fact, it is acknowledged as being of utmost importance to investigate the effect of PUFA supplementation in ruminants’ diets on mammary gland gene networks related to milk synthesis and udder homeostasis, aiming to develop targeted and tailor-made strategies to produce functional dairy products fortified with PUFAs.

The advent of advanced lab techniques and software tools initiated the -omics technologies, such that Nutrigenomics, as a sub-field of nutritional study, refers to the examination of food and its constituents on the regulation of gene expression [10] and the subsequent effects on the metabolic level [11]. Understanding the interactions between a genome and nutrients may potentially promote customized diets for specific needs [12], such as the development of functional dairy products. 

In this review, the intention is not to present the established knowledge of the field of Nutrigenomics in its entirety, but rather to place an emphasis on the mammary gland’s gene expression as related to fat synthesis under the implementation of dietary strategies focusing on the development of functional dairy products enriched with PUFAs and the potential of several dietary bioactive compounds to alleviate the oxidative balance and the homeostasis of the mammary gland. 

## 2. The Mammary Gland: Gene Network and Milk Fat Synthesis 

**Figure 1:**
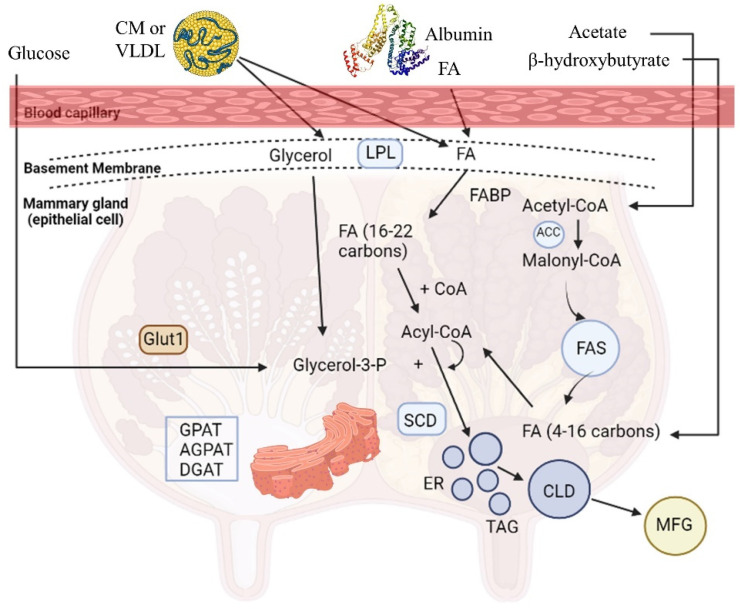
Milk fat synthesis in the ruminant mammary epithelial cell [15]. Abbreviations: ACC = acetyl-CoA carboxylase; AGPAT = acyl glycerol phosphate acyl transferase; CD36 = fatty acid transferase (CD36); CLD = cytoplasmic lipid droplet; CoA = coenzyme A; CM = chylomicron; DGAT = diacylglycerol acyltransferase; ER = endoplasmic reticulum; FA = fatty acid; FABP = fatty acid binding protein; FAS = fatty acid synthase; Glut1 = glucose transporter1; GPAT = glycerol-3 phosphate acyltransferase; LPL = lipoprotein lipase; MFG = milk fat globule; SCD = stearoyl-CoA desaturase; TAG = triglyceride; VLDL = very-low-density lipoprotein.

Milk is a highly nutritious product, crucial for the growth and health of neonates, which is synthesized by the mammary epithelial cells (MECs) and secreted by the mammary glands of mammals. The mammary gland synthesizes a variety of milk components, including proteins (casein 80% and whey protein 20%), carbohydrates (mainly lactose), coated lipid droplets, water, and ions [13]. Milk fat triglycerides (TAGs; 98% of total milk lipids) contain SFAs (C4:0-C18:0), palmitoleic (cis-9-16:1), oleic (cis-9-18:1), trans-18:1, and linoleic acid (cis-9, cis-12-18:2) [14]. Milk TAGs have a dual origin; one source is the de novo synthesis of fatty acids (FAs) produced by the MECs of the mammary gland through acetyl-CoA carboxylase (ACC) and fatty acid synthase (FAS) from circulating acetate and β-hydroxybutyrate (BHBA), a process which results in the formation of short- (SCFAs) and medium- (MCFAs) chain FAs (C4:0 to C16:0) that constitute almost half of the percentage of the milk FAs (Figure 1). The other source is FAs taken up by the mammary gland from blood circulation coming from the intestinal absorption of dietary and microbial FAs. Particularly, the long-chain FAs (LCFAs) (≥C18:0), along with 50% of C16:0 and a small proportion of C14:0, derive from blood plasma circulation released by lipoprotein lipase (LPL) from TAGs circulating in chylomicrons (CM) or very low-density lipoprotein (VLDL), or from the plasma NEFA bound to albumin [15] (Figure 1). Then, FAs are carried into the cells by FA translocase (CD36), FA transport protein (FATP), and FA binding protein (FABP) relative to the acyl-CoA synthetase or free FA receptors (FFARs). Subsequently, FAs are bound to FA binding proteins (e.g., FABP4) and subjected to different outcomes, including oxidation in mitochondria or esterification and storage in lipid droplets [8]. As for esterification, MCFAs are not esterified in cells which have an improper number of TAGs, while the storage of LCFAs does not have such a requirement [16].

De novo lipogenesis appears to be an important topic when the development of functional dairy products is concerned since there is ample evidence showing that PUFA supplementation in ruminants’ diets can severely downregulate these gene networks [8]. The three key enzymes that affect the de novo synthesis pathway of FAs are acyl-CoA synthetase short-chain family member 2 (ACSS2), ACC, and FAS. First, ACSS2 is the activator of acetic acid and BHBA in MECs, which are transformed to acetyl-CoA after connecting to pyruvate. The ACC enzyme carboxylates acetyl-CoA for the formation of malonyl-CoA, MCFAs, or LCFAs by FAS action in the cytosol [17] (Figure 1). Other enzymes, such as ATP-citrate lyase (ACL), FA elongase (ELOVL), and stearoyl-CoA desaturase (SCD), have a significant role in de novo synthesis [8]. ELOVL and SCD cooperate to synthesize MUFAs or PUFAs. FA desaturase enzymes (e.g., SCD) catalyze the conversion of SFAs to MUFAs or PUFAs, while FA elongases (ELOVL1 to ELOVL7) the inhibiting step of the LCFA elongation cycle in ruminants. SCD (Figure 1) is an enzyme from the endoplasmic reticulum (ER) enzyme, called a Δ9-desaturase because of a cis-9 double bond on the fatty acyl-CoA substrates, mostly from C14:0 to C19:0 [18]. SCD mostly uses stearic acid (C18:0) as a substrate for cis-9-18:1 synthesis, the main UFA found in milk triacylglycerol, implicated in the regulation of milk fat globules’ fluidity in the mammary gland [19]. Also, SCD can convert trans-vaccenic acid (C18:1 trans-11), an intermediate of biohydrogenation, to CLA cis-9, trans 11 [20].

As mentioned previously, the operation of TAGs is underscored to fully explain the mechanism of TAG synthesis in the smooth ER (Figure 1) and the coordination via subsequent reactions by the products of glycerol-3-phosphate acyltransferase (*GPAT*), lipin 1 (*LPIN1*), and diacylglycerol acyltransferase 1 (*DGAT1*) genes. The first step is the acylation of glycerol-3-phosphate (glycerol-3-P) for lysophosphatidic acid (LPA) formation, catalyzed by the GPAT enzyme. LPA acyltransferase (AGPAT) transfers a FA to LPA in order to produce phosphatidate (PA). The PA is converted to diacylglycerol (DAG) by the lipin, an ER enzyme, and lastly, from DAG to TAG by the DGAT enzyme [15,21]. TAGs in small lipid droplet formation are enveloped by the ER plasma membrane and escape from the MECs in larger lipid droplets through the apical membrane into the alveoli lumen and mammary gland duct, and they dissociate from the cell (Figure 1). 

The detection of transcription factors (TFs) that perceive the presence of lipids, notably FA, along with the comprehension of FA interference in several pathways and functions in cells, has shed light on the biological roles of FAs [22]. Overall data suggests that *SCD* is a key gene for milk fat synthesis, as it is the one with the highest gene expression, while the TF of sterol regulatory element binding transcription factor 1 (*SREBF1*), as well as the collaborative action of proliferator activated receptor γ (*PPARγ*), PPARγ coactivator 1 alpha (*PPARγC1A*), and insulin-induced gene 1 (*INSIG1*), have a crucial role in this process [23]. Milk fat synthesis in ruminant MECs is summarized in Figure 1, while the key genes involved in this process are listed in Table 1.

## 3. PUFA Dietary Supplementation Modifies a Complex Gene Network in Ruminants’ Mammary Glands

PUFA inclusion in ruminants’ diets appears to be an effective strategy for improving dairy products’ nutritional quality since beneficial milk FAs, namely cis-9-18:1, vaccenic, conjugated linoleic acid (CLA), and α-linolenic acid (ALA), can be increased [24], and they are associated with the prevention or decrease of the risk of chronic diseases in humans [25,26]. There is a noteworthy suggestion that UFA supplementation in ruminants’ diets downregulates mRNA abundance of *ACC*, *FAS*, and *SCD1*, as well as *SREBF1* and *PPARγ* [27,28]. In this section, a summary of studies concerning the effect of PUFA supplementation in ruminants’ diet in key genes’ expression in the mammary gland, which participate in de novo lipogenesis (*FAS*, *ACC*, *ACSS*, and *ACSL*), FA uptake (*LPL*, *CD36*), FA desaturation (*SCD1*, *FADS*), long-chain FA elongation (*ELOVL5* and *ELOVL6*) and transport (*FABP3* and *FABP4*), TAG synthesis (*GPAT*, *AGPAT*, *DGAT1*, *LPIN1*, and *GPAM*), as well as transcriptional regulation (*SREBF1*, *PPARγ*, and *INSIG1*) and lipid droplet formation (*BTN1A1* and *XDH*), are presented (see Table 2). The PUFA dietary treatments cited subsequently focus on the inclusion of fish oil, rich in EPA (eicosapentaenoic acid, 20:5 n-3) and DHA (docosahexaenoic acid, 22:6 n-3), plant-derived oils, and seeds such as soybean, sunflower, safflower (*Carthamus tinctorius* L.), rapeseed, canola, and linseed, rich in linoleic acid or α-linolenic acid, respectively, as well as the infusion of trans-10, cis-12 CLA in cows, ewes, and goats (Table 2). 

Beginning with fish oil, its supplementation in dairy cows’ diets downregulated the expression of *SCD1*, *FAS*, *ACC*, and *LPL* [29]. On the contrary, in another study, the inclusion of fish oil or DHA-rich microalgae in dairy cows’ diet reduced *SREBF1* expression in the mammary tissue, but this reduction was inadequate to substantially alter the gene expression of enzymes implicated in milk fat synthesis [30]. Vargas-Bello-Pérez et al. [31] investigated the inclusion of fish and soybean oil in dairy cows’ diets and found that fish oil reduced the expression of *ACC*, *PPARγC1*, *LPIN1*, and *FABP3* in the mammary gland, while soybean oil downregulated *ACC*, *INSIG1*, *DGAT1*, and *LPIN1*. Other authors found that the supplementation with fish and soybean oil [32], as well as with marine DHA-rich algae, along with sunflower and linseed oil [33], downregulated the expression of *SREBF1*, *SCD1*, *FAS*, and *LPL* in the bovine mammary gland. In dairy ewes, fish oil supplementation tended (*p* < 0.10) to reduce the mRNA abundance of *ACSS2*, *FAS*, *LPIN1*, *FADS2*, and *INSIG1*, while a downregulation observed in *ACC* and *ACSS1* genes’ expression was not significant [34]. Moreover, the expression of *ACC*, *ACSL1*, *ACSS1*, *ACSS2*, *ELOVL6*, *FADS2*, *AGPAT2*, and *LPIN1* genes was reduced in the ovine mammary gland under fish oil-induced milk fat depression (MFD) [35]. *ACC*, *ACSS1*, and *AGPAT6*, as well as *SREBF1* gene expression, was also decreased by fish oil inclusion in ewes’ diet, whereas *FABP3*, *LPL*, *SCD1*, and *INSIG1* expression tended to be decreased [36]. Furthermore, in dairy goats, the mRNA abundance tended to decrease for *SCD1* (*p* < 0.10) in the mammary gland when they were fed with fish and linseed oil simultaneously, while *SREBF1* and *PPARγ* were upregulated [37]. On the contrary, fish and linseed oil supplementation had no effect on *FAS* and *G6PDH* expression. A study examining the effect of fish oil inclusion in different goats’ tissues demonstrated a decline in the expression of *FAS*, *SCD1*, and *FADS2* only in the liver, but not in the mammary or omental adipose tissue [38]. Another study also showed that the inclusion of fish and soybean oil in dairy cows’ diets had no significant difference in the expression of either *SREBF1* or *PPRAγ* [39]. 

Summarizing the above results, it seems that, even though the inclusion of fish oils in ruminants’ diets is an effective strategy for increasing PUFA content in milk and therefore offering a functional product for consumers, a danger to the animals’ productivity is lurking as fish oils are associated with MFD syndrome. The development of MFD seems to be related to the downregulation of key genes involved in mammary glands’ lipogenesis, notably in de novo synthesis; a reduced expression of these genes was observed in most studies. In addition, the animal species per se may constitute a key factor affecting gene expression in the mammary gland, as goats, compared to cows and ewes, were less affected by fish oil supplementation in their diet. 

Apart from fish oil, other studies exclusively examined the effect of plant-derived oils and seeds in key genes’ expression in the mammary gland, and they mostly found a tendency for reduction in the mRNA abundance of those genes (Table 2). Specifically, the inclusion of sunflower oil in dairy cow diets reduced *ACC*, *FAS*, *GPAT*, and *AGPAT* mRNA abundance, while *SCD1* and *LPL* tended to be decreased (*p* < 0.08) [40]. Regarding dairy goats, supplementation with sunflower oil or linseed oil following hay-based diets caused an inhibition in mammary *SCD1* and *LPL* activity [41]. Additionally, inclusion of linseed or safflower oil downregulated the expression of *SREBF1*, *FAS*, and *ACSS1* significantly in the bovine mammary gland, while the decrease observed in *ACC* and *SCD1* was not statistically significant [42]. Lastly, the expression of *SCD1* was significantly decreased by the oil supplementation in the dairy cows’ diet, especially by soybean oil compared with rapeseed or linseed oil [43], and this result was associated with lower desaturase indexes, used for mammary SCD1 activity, observed in milk. On the contrary, rapeseed and sunflower oil supplementation had no effect on the mRNA abundance of either *LPL*, *ACC*, *FAS*, *SCD1*, *FABP3* and *FABP4*, and *XDH* [44,45] or *GPAM*, *DGAT1*, *CD36*, *INSIG1* [45], and *BTN1A1* [44] in bovine mammary tissue. The same pattern was observed in goats’ mammary tissue as the inclusion of sunflower oil and linseed oil in maize silage-based diets had no effect on the abundance of mRNA encoding for *LPL*, *ACC*, *FAS*, or *SCD1* [46]. Neither were alterations observed in the mRNA abundance of *ACC*, *FAS*, *LPL*, or *SCD1* in the mammary tissue of lactating beef cows under the effect of dietary linoleate–safflower seed oil [47]. On the contrary, formaldehyde-treated linseed and oleic sunflower oils in lactating goats’ diets decreased mammary *SCD1* mRNA, but no notable change was observed for *ACC* or *FAS* [48]. Instead, this research indicated that mammary *LPL* mRNA increased with oleic sunflower oil supplementation, but the activity of *G6PD* was not affected by the lipid supplements [48]. Recently, a study showed that dietary inclusion of linseed reduced the *SCD1*, *LPIN3*, *ELOVL5*, and *ELOVL6* genes’ regulation, while the mRNA abundance of *ACC*, *FAS*, *DGAT1*, *SREBF1*, and *FADS1* did not change significantly [49]. In contrast, an increase in the transcripts of other genes implicated in TAG synthesis, apart from *DGAT1*, such as *GPAT4*, *AGPAT1*, *AGPAT2*, and *AGPAT3*, was observed. The authors suggested that the inclusion of linseed reduced the overall metabolic activity of fat and protein, in contrast to carbohydrate, which increased, and as a result, the pathways of glycolysis, gluconeogenesis, and pentose phosphate were upregulated. On the other hand, inclusion of canola seeds in dairy cows’ diets did not alter the *SCD1* or *ACC* gene expression [50], nor did the addition of dietary long-chain FAs, provided by soybeans, have a significant effect on the mRNA abundance of *ACC* or *FAS* [51]. Lastly, supplementing goats’ diets with safflower or linseed oil increased the mRNA expression of *SCD1*, *ACC*, and *LPL* in the mammary tissue, but the mRNA expression of *FAS* was not affected by the oil supplementation [52]. 

To sum up, the effect of plant oil inclusion in ruminants’ diets seems to still be ambiguous as, in many studies, no significant effects were noted in the mRNA abundance of key genes in mammary tissue. It could be hypothesized that plant oils in comparison with fish oils are less capable of altering genes’ expression, and as a result, plant oils contribute to MFD to a lesser extent. This phenomenon could be attributed to the DHA and other very-long-chain PUFAs, such as EPA and DPA (docosapentaenoic acid, 22:5 n-3), found in fish and marine oils, which inhibit the rumen biohydrogenation of vaccenic acid (C18:1 trans-11) to C18:0, and consequently plenty of intermediates (such as trans-10, cis-12 CLA and trans-10 18:1) accumulate in the rumen and act as lipogenesis inhibitors in the mammary gland. It could be suggested that a combination of fish oils, for modifying the biohydrogenation process in the rumen, and plant oils, as a substrate for C18:1 trans-11 formation, in ruminants’ diets is a promising strategy for increasing cis-9, trans-11 CLA content in milk fat. However, there is a need for further investigation as the effect of plant oils depends on their composition and origin, as well as on the experiments’ duration (rumen microbiome adaptation).

Three studies examining the infusion of trans-10, cis-12 CLA in dairy cows indicated a reduced expression of *SREBF1* and *FAS* [53,54,55], along with *LPL* [54], *ACC* [55], or *SCD1* [53]. A similar pattern was also observed in trans-10, cis-12 CLA-induced MFD in lactating ewes, which resulted in *FAS*, *ACC*, *ACSS2*, *ACSS3*, and *FADS2* downregulation [56]. Hussein et al. [57], also experimenting with CLA-induced MFD in lactating ewes, incorporated a lipid-encapsulated CLA supplement containing cis-9, trans-11 and trans-10, cis-12 CLA isomers, and they found that the mRNA abundance of *ACC*, *FAS*, *LPL*, *SCD1*, *AGPAT6*, *SREBF1*, and *INSIG1* was reduced. Moreover, a comparative study of the inclusion of fish oil- or trans-10, cis-12 CLA in the mammary mRNA abundance of some lipogenic genes and TFs showed that fish oil downregulated the expression of *ACSS2*, *FAS*, and *LPIN1*, while *ACC* had no significant difference. Additionally, *LPL* and *LPIN1* were lower with fish oil than with CLA, whereas *PPARγ* was higher in ewes receiving CLA. Furthermore, treatment with CLA upregulated *GPAT4*, while the abundance of *FABP3*, *SCD1*, *GPAM*, and *SREBF1* did not variate [58]. Tsiplakou et al. [59] evaluated the key genes’ expression in sheep and goats’ mammary glands under two feeding strategies and found that the mRNA abundance of *ACC* and *FAS* was lower in sheep compared to goats when animals were fed on either a group or an individual basis. In contrast, *LPL* mRNA levels in the mammary gland did not vary between sheep and goats fed on a group basis. However, the mRNA level of *SCD1* was higher in sheep compared to goats, independent of the way they received their diets (group or individual), linked to the higher concentration of cis-9 trans-11 CLA content in sheep milk fat compared with that of goats.

Overall, fish and plant oil supplementation in ruminants’ diets confirms the initial statement that PUFA supplementation in ruminants’ diet downregulates, in most studies, the expression of key genes involved in the pathways of de novo synthesis (e.g., *ACC* and *FAS*) and FA activation (e.g., *ACSS1*, *ACSS2* and *ACSL1*), FA uptake and transport (e.g., *LPL* and *FABP3*), FA desaturation (e.g., *SCD1* and *FADS2*), TAG synthesis (e.g., *AGAPT6* and *LPIN1*), FA elongation (e.g., *ELOVL6*), as well as TFs (e.g., *SREBF1*, *INSIG1*, and *PPARγ*) involved in their regulation. However, according to the aforementioned literature, it seems that genes involved in de novo synthesis (e.g., *FAS* and *ACC*) have more plasticity compared to those implicated in other pathways, such as desaturation (e.g., *SCD1*). Additionally, the alterations of the former gene expressions in dairy cows’ mammary glands under the impact of such dietary strategies depicted higher consistencies compared with those observed in small ruminants. The TFs were also modified by PUFA supplementation in most cases. Generally, the synchronized downregulation in key gene expression, observed in many studies in dairy ruminants [60,61,62], encourages the idea that there is a central regulator, probably *SREBF1* or *PPARγ*, of milk fat synthesis. *SREBF1* downregulation is frequently detected during MFD induced by marine lipid supplementation [32,33,36,39] or CLA [55,57]. The reduction of *SREBF1* mRNA abundance has shown an inhibition of the SREBP1 signaling pathway and its target genes [15,63], which partly explains the negative effects of dietary oil supplementation on many genes’ expression, including *ACC*, *FAS*, *LPL*, *FABP3*, and *SCD1*. *PPARγ* could partially regulate the expression of *SREBF1*, which in turn could affect the activity of *PPARγ* by increasing the production of its natural agonists [62]. The downregulation of *SREBF1* is also correlated with *INSIG1* reduction, as *INSIG1* is known to be involved in *SREBF1* activation to its mature form [64]. The capability of diet to alter the mRNA abundance of *SREBF1* and *INSIG1* has been previously reported by several researchers [32,36,57,65]. 

The intense inhibition of de novo synthesis and the resulting milk fat depression (MFD) observed in many cases could be attributed to the reduction of SCFAs as this reduction can reach up to 60% [29]. These effects can be attributed to the synthesis of specific rumen biohydrogenation intermediates associated with the ruminal trans-10 pathway that exerts antilipogenic effects in bovines [60]. The downregulation of the genes, implicated in de novo synthesis, has been suggested as decreasing the milk fat percentage. However, in the study of Ahnadi et al. [29] the milk fat content was significantly associated only with *ACC*, *FAS*, and *SCD1* gene expression, but not with that of *LPL*. On the contrary, provided data in ruminants suggest that the feeding strategies which increase LCFA in the produced milk, e.g., the inclusion of plant oils [15,66], do not reduce the mRNA abundance of *LPL*. Besides, the downregulation of *LPL* has been related to a reduction in milk fat synthesis [32,33,36,57]. Concerning *SCD1* gene expression, its nutritional regulation involves complex interactions between dietary factors and regulatory events which have not been intensively described [67]. Few studies have shown that *SCD1* is downregulated when induced-MFD diets are provided to ruminants, such as those containing fish and plant oils [29,33,40], as well as CLA [53,55,57]. A possible explanation for that reduction might be the increases observed in EPA and trans-10, cis-12 CLA [67,68] and the reduction of cis-9 18:1 in milk, which is related to milk fat fluidity and to the negative effect of marine lipids on mammary lipogenesis [60,69,70]. However, in other studies, the inclusion of fish and soybean oils causing MFD is associated with a lack of alteration or even an increase in *SCD1* mRNA levels [32,39,58,71]. Thus, there is a suggestion that these variations in *SCD1* expression could be due to the nutritional regulation of its expression [67], depending on the origin and doses of the supplemented oils, as well as the period of adaptation to the treatments. Apart from *SCD1* regulation, Conte et al. [49] showed that the inclusion of linseed resulted in downregulation of *FADS1* and *FADS2* expression related to the increase in milk PUFAs due to diet or ruminal activity (CLA) and the reduction in very-long-chain PUFAs (such as EPA and DHA). According to Mele et al. [72], these two genes (*FADS1* and *FADS2*) engage in the synthesis of EPA and DHA by adding double bonds at the Δ5 and Δ6 positions. 

Finally, among the genes involved in the last steps of milk fat synthesis, FA esterification to glycerol and the secretion of milk fat globules, *DGAT1* abundance does not vary significantly during MFD [36,39,57,71], perhaps due to its known post-transcriptional regulation [15]. On the contrary, the downregulation of *AGPAT6* observed in many studies shows that there is a kind of inhibition of de novo lipogenesis, probably due to its greater affinity for short- and medium-chain SFAs [15].

**Table 2:** Selected studies presenting the impact of PUFA-rich diets and supplements on mammary gland gene network that regulate fat synthesis.

Method	Diet Supplement	Level/Detail	Effect	Reference
Dairy cows				
In vivo	Fish oil	3.7% of DM unprotected fish oil, or 1.5% or 3.0% of DM glutaraldehyde-protected microcapsules of fish oil	↓ *SCD1*, *FAS*, *ACC*, *LPL*	[29]
In vivo	Fish oil or DHA-rich microalgae	200 g/d	↓ *SREBF1* No difference in gene expression of enzymes implicated in milk fat synthesis	[30]
In vivo	Soybean oil or fish oil	2.9% of DM unrefined soybean oil or 2.9% DM of fish oil manufactured from salmon oil	Soybean oil: ↓ *ACC*, *INSIG1*, *DGAT1*, *LPIN1* Fish oil: ↓ *ACC*, *PPARγC1*, *LPIN1*, *FABP3*	[31]
In vivo	Short-term administration of trans-10, cis-12 CLA, and a low forage/high oil (LF/HO) diet	10 g/d infusion of trans-10, cis-12 CLA and LF/HO diet, including 3.0% soybean oil and 1.5% fish oil	↓ *SREBF1*	[32]
In vivo	Silage-based diets supplemented with palm fat, linseed oil plus DHA-rich algae, or sunflower oil plus DHA-rich algae	3.1% of the basal diet DM of rumen-stable fractionated palm fat, a mixture of linseed oil (2.7% of the basal diet DM) plus DHA-rich algae (0.4% of the basal diet DM), or a mixture of sunflower oil (2.7% of the basal diet DM) plus DHA-rich algae (0.4% of the basal diet DM)	↓ *SREBF1*, *SCD1*, *FAS* No difference in *LPL*, *GPAM*	[33]
In vivo	Fish and soybean oil	3.5% of DM (1% fish oil and 2.5% soybean oil)	No difference in *SREBF1* or *PPRAγ*	[39]
In vivo	Sunflower oil	1% of DM sunflower oil	↓ *ACC*, *FAS*, *GPAT*, *AGPAT* while ↓ *SCD1*, *LPL* (tendency, *p* < 0.08)	[40]
In vivo	Linseed or safflower oil	5% linseed or safflower oil on DM	↓ *SREBF1*, *FAS*, *ACSS1* ↓ *ACC*, *SCD1* (not significant)	[42]
In vivo	Soybean, rapeseed, or linseed oil	2.7% soybean, rapeseed, or linseed oil on DM basis, or 2.7% of a 1:1:1 mixture of the 3 oils	↓ *SCD1* (especially by soybean oil)	[43]
In vivo	Rapeseed or sunflower oil	130 g/d of oil from whole, intact rapeseeds in a HF diet (F:C = 64:36), or 130 g/d of sunflower oil in a LF diet (F:C = 43:57)	No difference in *LPL*, *ACC*, *FAS*, *SCD1*, *FABP3*, *FABP4*, *XDH*, *BTN1A1*	[44]
In vivo	Rapeseed or sunflower oil	13.9% of DM whole, intact rapeseeds in a HF diet (F:C = 65:35) or 4% of DM sunflower oil in a LF diet (F:C = 46:54)	No difference in *LPL*, *ACC*, *FAS*, *SCD1*, *FABP3*, *FABP4*, *XDH*, *GPAM*, *DGAT1*, *CD36*, *INSIG1*	[45]
In vivo	Linoleate–safflower seed	low-fat control supplement (64.2% cracked corn, 32.1% safflower seed meal, and 3.7% liquid molasses) fed at 0.35% of BW daily (DM basis) or a cracked, high-linoleate safflower seed supplement (94.0% cracked, high-linoleate safflower seeds and 6% liquid molasses) at 0.23% of BW daily (DM basis)	No difference in *ACC*, *FAS*, *LPL*, *SCD1*	[47]
In vivo	Canola seeds	4.8% of DM canola meal, 3.3% of DM unprotected canola seeds plus 1.5% canola meal, or 4.8% of DM formaldehyde-protected canola seeds	No difference in *SCD1*, *ACC*	[50]
In vivo	Infusion of trans-10, cis-12 CLA	13.6 g/d	↓ *SREBF1*, *FAS*, *SCD1*	[53]
In vivo	Infusion of trans-10, cis-12 CLA	7.5 g/d	↓ *SREBF1*, *FAS*, *LPL*	[54]
In vivo	Infusion of trans-10, cis-12 CLA	10 g/d	↓ *SREBF1*, *FAS*, *ACC*	[55]
Dairy ewes				
In vivo	Fish oil	20 g of fish oil/kg of DM	↓ *ACSS2*, *FAS*, *LPIN1*, *FADS2*, *INSIG1* (tendency, *p* < 0.10) ↓ *ACC*, *ACSS1* (not significant)	[34]
In vivo	Fish oil	Transcriptome analysis in milk somatic cell (MECs) of ewes suffering from fish oil-induced MFD	↓ *ACC*, *ACSL1*, *ACSS1*, *ACSS2*, *ELOVL6*, *FADS2*, *AGPAT2*, *LPIN1*	[35]
In vivo	Fish oil	17 g of fish oil/kg of diet DM	↓ *ACC*, *ACSS1*, *AGPAT6*, *SREBF1* ↓ *FABP3*, *LPL*, *SCD1*, *INSIG1* (tendency, *p* < 0.10)	[36]
In vivo	Linseed	20% of DM linseed panel	↓ *SCD1*, *LPIN3*, *ELOVL5*, *ELOVL6* ↑ *GPAT4*, *AGPAT1*, *AGPAT2*, *AGPAT3* No difference in *ACC*, *FAS*, *DGAT1*, *SREBF1*, *FADS1*	[49]
In vivo	Trans-10, cis-12 CLA-induced MFD	10 g of a rumen-protected CLA product/kg of diet DM	↓ *FAS*, *ACC*, *ACSS2*, *ACSS3*, *FADS2*	[56]
In vivo	Lipid-encapsulated CLA supplement containing cis-9, trans-11 and trans-10, cis-12 CLA isomers	15 g/d	↓ *ACC*, *FAS*, *LPL*, *SCD1*, *AGPAT6*, *SREBF1*, *INSIG1*	[57]
In vivo	Comparative study between the inclusion of fish oil- or trans-10, cis-12 CLA	2.4% of DM fish oil or 1% of DM a commercial product rich in trans-10,cis-12 CLA	Fish oil: ↓ *ACSS2*, *FAS*, *LPIN1*, no difference in *ACC* CLA: ↑ *GPAT4*, no difference in *FABP3*, *SCD1*, *GPAM*, *SREBF1* *LPL*, *LPIN1* were lower in fish oil than in CLA *PPARγ* was higher in ewes receiving CLA	[58]
Dairy goats				
In vivo	Fish and linseed oil	530 g/day of extruded linseeds (EL) or 340 g/day of extruded linseeds plus 39 g/day of fish oil (ELFO)	ELFO: ↓ *SCD1* (tendency, *p* < 0.10) ↑ *SREBF1*, *PPARγ* No difference in *FAS*, *G6PDH*	[37]
In vivo	Fish oil	90 g/day of sunflower oil and fish oil (2:1) plus additional starch	↓ *FAS*, *SCD1*, *FADS2* only in liver tissue (not in mammary and omental adipose tissue)	[38]
In vivo	Sunflower oil or linseed oil following hay-based diets	55 g/kg diet DM sunflower oil or linseed oil	Inhibition in mammary *SCD1* and *LPL* activity	[41]
In vivo	Sunflower oil and linseed oil in maize silage-based diets	6.1% of diet DM sunflower oil or 6.2% of diet DM linseed oil	No difference in *LPL*, *ACC*, *FAS*, *SCD1*	[46]
In vivo	Formaldehyde-treated linseed or oleic sunflower oil	11.2% of DM intake formaldehyde-treated linseed or 3.5% of DM intake oleic sunflower oil	↓ *SCD1* No difference in *ACC*, *FAS*, *G6PD* ↑ *LPL* with oleic sunflower oil	[48]
In vivo	Soybeans	22% of DM soybeans	No difference in *ACC*, *FAS*	[51]
In vivo	Safflower or linseed oil	50 g/kg of TMR DM safflower oil or 50 g/kg of TMR DM linseed oil	↑ *SCD1*, *LPL* ↑ *ACC* (with safflower oil) No difference in *FAS*	[52]
Dairy ewes and goats				
In vivo	Two feeding strategies: group or individual basis	Basic diets	Lower *ACC* and *FAS*, but higher *SCD1* in ewes compared to goats in both feeding strategies No difference in *LPL* between ewes and goats fed on a group basis	[59]

↓ = decrease, ↑ = increase.

## 4. Bioactive Nutrients: An Ally of Mammary Gland Homeostasis

The inclusion of dietary FAs in the diet of ruminants, apart from the beneficial role in the development of dairy products enriched with bioactive molecules, such as omega-3 FAs (functional foods), can cause some negative effects, such as oxidative stress and consequently homeostatic imbalances on the mammary gland. Therefore, it is acknowledged as being of utmost importance to both examine the potential of various feed antioxidant components to counteract these effects and to comprehend the mechanisms underlying these features, will be valuable as a contribution to the alleviation of such phenomena. 

Cells produce both ROS (reactive oxygen species) and RNS (reactive nitrogen species), as part of their physiological metabolism in order to regulate their homeostasis, as signaling molecules [73]. However, ROS overproduction can induce severe oxidative imbalance by impairing proteins, lipids, or even DNA, causing a situation broadly known as oxidative stress [73], which has a highly negative relation with the performance, health, and welfare of ruminants [74,75]. PUFAs are generally prone to non-enzymatic (autoxidation and photooxidation) and enzymatic oxidation [9]. During PUFA oxidation, various metabolites are generated which induce oxidative stress mainly by increasing the concentration of lipid peroxidation aldehydes, such as malondialdehyde (MDA), and activating superoxide anion generators, such as NADPH oxidases [9], affecting the cellular homeostasis of the mammary gland. The source of PUFAs, as well as their dietary inclusion levels, can influence the oxidative status of target tissues. Therefore, dietary supplementation of antioxidant compounds along with PUFA-enriched rations may have the potential to lessen the appearance of oxidative stress incidence and consequently mammary gland homeostasis. Indeed, the supply of antioxidant compounds, namely whole sesame seeds or sesame meal combined with vitamin E and selenium [76,77] delivered some promising results for minimizing oxidative stress along with inflammation in ruminants [78,79]. Apart from the contribution of antioxidant compounds in ruminants’ nutrition on the animal level, there is a greater need for fully understanding the changes and influence occurring on the molecular level as part of the expression of specific genes in the mammary gland.

### 4.1. NFE2L2 Factor and Its Significance in Antioxidant Cellular Defense

In whatever manner, the redox balance can be manipulated through nutrition by stimulating the expression of specific genes for antioxidant properties. A crucial mechanism for preventing the hazardous aftermath of oxidative stress is the *NFE2L2* pathway, activated under stressful conditions, supplying oxidant compounds, or under inflammation [80]. The antioxidant defense and cellular response appear to be controlled by a set of TFs, including nuclear factor erythroid 2-like 2 (*NFE2L2*), nuclear factor-kappa B (*NFKB*), *PPARs*, and *PPARγC1A*. The Kelch-like epichlorohydrin-associated protein 1 (*KEAP1*)–*NFE2L2*–antioxidant response element (ARE) signaling pathway significantly preserves cellular homeostasis through the regulation of genes related to cellular redox balance [81]. The activation of *NFE2L2* reflects on the complex formed by *KEAP1* and *CUL3* [82]. Concisely, under homeostatic conditions, *KEAP1* attaches with *NFE2L2*, contributing to *NFE2L2* degradation, and thus overcoming its activation [83]. Conversely, the overproduction of ROS results in the unbinding of *KEAP1* and *NFE2L2*, subsequently leading to *NFE2L2* activation and an increase in glutathione (GSH) production [84]. *NFE2L2*, as a TF, belongs to the leucine zipper family and can be activated by antioxidant elements located in the regulatory regions of many antioxidant genes [85]. After, *NFE2L2* is released from *KEAP1* and transferred to the nucleus, where it stimulates the transcription of genes associated with the antioxidant response [86,87]. More specifically, it appears to have a crucial role in the mammary gland antioxidant status, through regulating its mRNA abundance [88]. Additionally, in vitro studies of cultured MECs showed that, after the activation of the *NFE2L2* pathway by several antioxidant compounds, the expression of antioxidant genes was stimulated [89]. However, not only nutrients [90] but also the existence of ROS can activate the *NFE2L2* pathway [91]. Nonetheless, the target genes of *NFE2L2*, although well-established in monogastric animals, have not been fully confirmed in ruminants. Nevertheless, there is a need for more precise experimental approaches and new techniques to further understand the mechanism of this TF and its dynamic in the lessening of oxidative stress [92].

### 4.2. Nutrients Affect Homeostatic Mammary Gland Gene Networks 

According to the accessed literature, plant compounds; AAs, such as methionine (Met) [93]; trace minerals and vitamins [79,94]; and plant bioactive compounds [95] seem to efficiently contribute to lessening the burden of oxidative stress (Table 3). Aside from that, the impaired antioxidant status can cause severe damage to the immune system; thus, nutrition research also targets the mechanisms regulating gene expression on the immune system, as has been shown by several studies that will be presented in the following section. To begin with, AAs, apart from contributing to protein synthesis, may effectively regulate various critical metabolic processes in the mammary gland [93]. So, the concept behind supplying animal diets with AAs with known antioxidant protection is to alleviate oxidative cellular damage [96,97], especially during the peripartum period. In addition, AAs can be metabolized by ruminants for creating sub-products, for instance, Met, to synthesize sulfur-containing antioxidants such as GSH [93]. Moreover, Arg has a crucial role in both innate and acquired immunity by modifying the activity, proliferation, and apoptosis of immune cells via the mTOR pathway [98]. In detail, Met enhanced antioxidant protection against heat stress in bovine MECs in vitro [99,100]. In another study by Han et al. [88], methionine upregulated the mRNA abundance of genes that control antioxidant mechanisms. More specifically, significant upregulations of *NFE2L2* target genes such as *GCLC*, *GCLM*, *GPX1*, *GSR*, *ME1*, *FECH*, *FTH1*, and *NQO1* were found. In contrast, the supply of Met had no effect on *HMOX1*, *TXN*, *GSTM1*, *TALDO1*, *BCL2*, or *PIR*. Additionally, the expression of *NFE2L2*, *CUL3*, *NFKB1*, and *MAPK14* was upregulated. As a result, the research group indicated that Met has a significant contribution to alleviate oxidative stress by regulating GSH metabolism in MECs.

In addition to the well-known importance of trace elements and vitamins as essential nutrients, they have also immunoregulatory and antioxidant properties. Deficiencies in these kinds of micronutrients are strictly associated with various disorders such as mastitis [101]. The mechanism that underscores the contribution of micronutrients to health is usually caused by their antioxidant features [102]. Selenium (Se) is a naturally derived antioxidant, easily supplied through feed. Since it is an essential component of 25 animals’ selenoproteins, it operates as a fundamental regulator for their activity and expression [103]. One of them is glutathione peroxidase (GPX), crucial for the antioxidant defense of cells and tissues. In a study by Sun et al. [104], the organic form of Se (hydroxy-selenοmethionine, HMSeBA, and selenomethionine, SeMet) resulted not only in higher GPX activity, but also in the enhanced mRNA abundance of *GPX3*, as compared to the sodium selenite (SS) form in bovine MECs. However, no effect was noted for *CAT* and *SOD* gene expression. Moreover, Han et al. [105], in an experiment with nano-selenium (nano-Se) and SS supplementation in dairy cows, found that dietary nano-Se upregulated the mRNA expression of *GPX1*, *GPX2* and *GPX4*, as well as that of thioredoxin reductase 2 and 3, in the mammary gland, compared with the SS group. The common stage for both was the significance of the proper selection of Se source.

Bioactive compounds derived from plants are widely known as potential contributors to decreasing oxidative stress in ruminants [106]. In several experiments with the use of polyphenols, some promising results have been published. For instance, the treatment of bovine MECs with tea polyphenols (100 μg/mL) enhanced the protein abundance of *NFE2L2* and *HMOX1* [95]. Thoroughly, the co-treatment of cells with tea polyphenols and H_2_O_2_ increased the mRNA abundance of *NFE2L2*, *HMOX1*, and *NQO1* compared to H_2_O_2_ treatment. In the same manner, the co-treatment of cells with tea-polyphenols and H_2_O_2_ caused an upsurge in *BCL2*, but it significantly diminished *BAX*, *CASPASE3*, and *CASPASE9* mRNA abundance compared with the H_2_O_2_ treatment [95]. In the same experiment, the mRNA expression of *TNFA*, *IL6*, and *IL1B*, was decreased in the tea-polyphenols plus H_2_O_2_-treated group compared with the H_2_O_2_-treated group, along with a reduced apoptosis rate [95]. Therefore, Ma et al. [95] concluded that tea-polyphenols may effectively set up a favorable antioxidant by inhibiting the activation of *CASPASE3* and the expression of *BAX* and by strengthening the mRNA expression of *BCL2*. In addition, tea-polyphenols can lessen the inflammatory burden caused by ROS by inhibiting the expression of inflammatory cytokines. Moreover, resveratrol (50 µM) induced similar effects in bovine MECs challenged with H_2_O_2_ [89]. However, it is worth mentioning that the use of polyphenols extracted from green tea in vivo had no effect on oxidative stress in peripartal cows [107]. Considering the in vivo trials, it has been reported that the post-ruminal bioavailability of tea-polyphenols may play a critical role in their antioxidant impact [108].

Among plant compounds with known antioxidant properties are the plant lignans due to their polyphenolic content, such as those contained in flax. Flax hulls have been assessed for their influence upon some target genes in the mammary gland specific to the expression of antioxidant features [109]. In this experiment, the dietary inclusion of flax hull in cows increased the mRNA abundance of *CAT*, *GPX1*, and *SOD1*, but lessened that of *GPX3*, *SOD2*, and *SOD3* in the mammary gland [110]. Moreover, when flax hulls were combined with 500 g/d flax oil in cows’ diets, the mRNA levels of *CAT*, *GPX1*, *GPX3*, *SOD2*, and *SOD3* were decreased, unveiling the prooxidant effect of PUFAs (flax oil) [109]. In another experiment, the inclusion of flax meal in dairy cows’ diets gave some prominent results on the regulation of specific target genes and TFs in the mammary gland [110]. The *NFE2L2* mRNA abundance was linearly increased among the inclusion levels, while the *CAT*, *GPX1*, *GPX3*, *SOD1*, *SOD2*, *SOD3*, and *NFKB* genes remained unaffected [110]. Palin et al. [111] also performed an experiment on dairy cows, supplementing flax hulls, with or without flax oil, for the effect of this nutritional treatment upon the expression of lipogenic genes. The inclusion of flax hulls alone upregulated the mRNA abundance of *SREBPF1*, *FAS*, *LPL*, *PPARγ1*, *SCD*, and *ACC* [111]. The results of this study demonstrated that the lignans present in flax hulls not only have significant antioxidant potential, but they can also rebalance key lipogenic gene networks that are generally downregulated by dietary PUFA overload. 

Lastly, the inclusion of piper meal, which is rich in bioactive polyphenols, increased the mRNA abundance of *SOD1*, *SOD2*, and *SOD3*, and for *NFE2L2*, while it did not affect that of *CAT*, *GPX1*, *GPX2*, or *GPX3* in dairy goats [112]. Furthermore, dietary supplementation with piper meal affected the immune-transcriptional profile of goats’ mammary glands by downregulating the expression of *NFKB1* [112]. Thus, it could be concluded that polyphenolic compounds have both immune-oxidative properties.

**Table 3:** Selected studies presenting the impact of bioactive components and feed additives on MECs and the mammary gland gene network.

Animal Species	Experimental Method	Nutrition Tested	Level	Effect	Reference
Bovine MECs	In vitro	Tea-polyphenols (GTP), H_2_O_2_, combination	500 µM H_2_O_2_ 100 µg/mL GTP 100 µg/mL GTP plus 500 µM H_2_O_2_	GTP: ↓ *BAX*, *CASPASE3*, *CASPASE 9* ↑ *BCL2* GTP + H_2_O_2_ vs. H_2_O_2:_ ↑ *BCL2*, *NFE2L2*, *HMOX1* ↓ *BAX*, *CASPASE3*, *CASPASE9*, *TNFA*, *IL6*, *IL1B*	[95]
Bovine MECs	In vitro	-Organic Se source hydroxy-selenomethionine (HMSeBA) -Selenomethionine (SeMet) -Sodium selenite (SS)	0 20 nM HMSeBA 50 nM HMSeBA 100 nM HMSeBA 150 nM HMSeBA 100 nM SeMet 100 nM SS	HMSeBA: ↑ *GPX3* No difference in *CAT*, *SOD* MSeBA100, SeMet100 and SS100: No difference in *GPX3*, *SOD1*, *CAT*	[104]
Dairy cows	In vivo	Rumen-protected methionine	0.09% and 0.10% of DMI	↑ *GCLC*, *GCL*, *GPX1*, *GSR*, *ME1*, *FECH*, *FTH1*, *NQO1*, *NFE2L2*, *CUL3*, *NFKB1*, *MAPK14* ↓ *KEAP1*	[88]
Dairy cows	In vivo	Nano-selenium (nano-Se) Sodium selenite (SS)	0.30 mg Se/kg DM SS 0.30 mg Se/kg DM Nano-Se	↑ *GPX1*, *GPX2*, *GPX4* No difference in *GPX3*	[105]
Dairy cows	In vivo	Flax hulls	no flax hulls (CONT) 9·88% flax hulls (HULL) 500 g flax oil/d (COFO) 9·88% flax hulls and 500 g flax oil/d (HUFO)	HULL vs. CONT: ↑ *CAT*, *GPX1*, *SOD1* ↓ *GPX3*, *SOD2*, *SOD3* COFO vs. CONT: ↓ *CAT*, *GPX1*, *GPX3*, *SOD3* HUFO vs. CONT: ↓ *CAT*, *GPX1*, *GPX3*, *SOD2*, *SOD3*	[109]
Dairy cows	In vivo	Flax hulls	no flax hulls (CONT) 9·88% flax hulls (HULL) 500 g flax oil/d (COFO) 9·88% flax hulls and 500 g flax oil/d (HUFO)	HULL: ↑ *SREBPF1*, *FAS*, *LPL*, *PPARγ1*, *SCD*, *ACC* No difference in *PPARγ2* COFO: ↑ *FAS*, *LPL*, *ACC* ↓ *PPARγ2*, *SCD* HUFO: ↑ *ACC*, *PPARγ1* ↓ *LPL*, *SCD*	[111]
Dairy cows	In vivo	Flax meal (FM)	5% (5FM) 10% (10FM) 15% (15FM)	↑ *NFE2L2*	[110]
Dairy goats	In vivo	Piper meal	CPM diet (1.3% piper meal per kg dry matter)	↑ *SOD1*, *SOD2*, *SOD3*, *NFE2L2* ↓ *NFKB1*	[112]

↓ = decrease, ↑ = increase.

## 5. Further Prospects

Considering the aforementioned research evidence, on the path towards promoting functional dairy products enriched with biomolecules beneficial for human health, such as omega-3 FAs, unfavorable cellular conditions may occur. However, it could be concluded that there are certain gene biomarkers that are more firmly linked to lipogenesis in the mammary gland, while these genes are also variously affected by different dietary fatty acids (PUFA source). Additionally, it was also unveiled that PUFA levels, sources, and animal species appear to also have crucial importance considering the regulation of the mammary gland lipogenic network. On the other hand, ample evidence suggests that dietary antioxidant compounds, and especially polyphenols and flavonoids, can strengthen the immune-oxidative status of the mammary gland, and their simultaneous supplementation may alleviate any side effect caused by dietary PUFA overload. Future studies should evaluate the effect of PUFA-rich diets, and especially the combination of different PUFA sources, achieving a sustainable transfer efficiency as a strategy to develop functional dairy products. Simultaneously, bioactive compounds with prominent antioxidant potential capable of neutralizing PUFAs’ adverse effects on mammary gland oxidative status should be also assessed in vivo. 

## Figures and Tables

**Table 1:** Key genes involved in milk fat synthesis in ruminants [17].

Full Name	Key Gene	Function
Acyl-CoA synthetase short-chain family member 2	*ACSS2*	De novo synthesis of FA
Fatty acid synthase	*FAS*	
Acetyl-CoA carboxylase	*ACC*	
Fatty acid transferase (CD36)	*CD36*	LCFA uptake in blood
Solute carrier family 27-member 6	*SLC27A6*	
Acyl-CoA synthetase long chain family member 1	*ACSL1*	
Fatty acid binding protein 3	*FABP3*	LCFA transport and desaturation
Stearoyl-CoA desaturase 1	*SCD1*	
1-Acylglycerol-3-phosphate o-acyltransferase 6	*AGPAT6*	TAG synthesis
Diacylglycerol O-acyltransferase 1	*DGAT1*	
Lipin 1	*LPIN1*	
Glycerol-3-phosphate acyltransferase, mitochondrial	*GPAM*	
Perilipin 2	*PLIN2*	Lipid droplet secretion
Xanthine dehydrogenase	*XDH*	
Butyrophilin subfamily 1 member A1	*BTN1A1*	

## Abbreviations

Abbreviation	Word or phrase in full
AAs	amino acids
*ACC*	acetyl-CoA carboxylase
ACL	ATP-citrate lyase
*ACSL1*	acyl-CoA synthetase long chain family member 1
*ACSS2*	acyl-CoA synthetase short-chain family member 2
*AGPAT*	acyl glycerol phosphate acyl transferase; lysophosphatidic acid acyltransferase
ALA	α-linolenic acid
ARE	antioxidant response element
Arg	arginine
*BAX*	Bcl-2 associated X-protein
BCL2	B-cell lymphoma-2
BHBA	β-hydroxybutyrate
*BTN1A1*	butyrophilin subfamily 1 member A1
CAT	catalase
CD36	CD36 molecule; fatty acid transferase
CLA	conjugated linoleic acid
CLD	cytoplasmic lipid droplet
CM	chylomicrons
CoA	coenzyme A
*CUL3*	cullin-3
DAG	diacylglycerol
*DGAT*	diacylglycerol acyltransferase
DHA	docosahexaenoic acid
DNA	deoxyribonucleic acid
DPA	docosapentaenoic acid
ECM	extra-cellular matrix proteins
ELOVL	fatty acid elongase
EPA	eicosapentaenoic acid
ER	endoplasmic reticulum
FABP	fatty acid binding protein
*FADS*	fatty acid desaturase
FAs	fatty acids
*FAS*	fatty acid synthase
FATP	fatty acid transport protein
*FECH*	ferrochelatase
FFARs	free fatty acid receptors
FFAs	free fatty acids
*FTH1*	ferritin heavy chain 1
*G6PDH*	glucose-6-phosphate dehydrogenase
*GCLC*	glutamate-cysteine ligase catalytic subunit
*GCLM*	glutamate-cysteine ligase modifier subunit
Glut1	glucose transporter1
glycerol-3-P	glycerol-3-phosphate
*GPAM*	glycerol-3-phosphate acyltransferase, mitochondrial
*GPAT*	glycerol-3-phosphate acyltransferase
*GPX*	glutathione peroxidase
*GSH*	glutathione
*GSR*	glutathione-disulfide reductase
*GSTM1*	glutathione s-transferase mu 1
*HMOX1*	heme oxygenase 1
HMSeBA	hydroxy-selenomethionine
*IL*	interleukin
*INSIG*	insulin-induced gene
*KEAP1*	Kelch-like epichlorohydrin-associated protein 1
LCFAs	long-chain fatty acids
LPA	lysophosphatidic acid
*LPIN*	lipin
*LPL*	lipoprotein lipase
*MAPK14*	mitogen-activated protein kinase 14
MCFAs	medium chain fatty acids
MDA	malondialdehyde
*ME1*	malic enzyme 1
MECs	mammary epithelial cells
Met	methionine
MFD	milk fat depression
MFG	milk fat globule
mRNA	messenger ribonucleic acid
mTOR	mechanistic target of rapamycin
NADPH	nicotinamide adenine dinucleotide phosphate
NEFA	non-esterified FA
*NFE2L2*	nuclear factor erythroid 2-like 2
*NFKB*	nuclear factor-kappa B
*NQO1*	NAD(P)H dehydrogenase [quinone] 1
O_2_	oxygen
PA	phosphatidate
*PIR*	pirin
*PLIN2*	perilipin 2
PPARs	proliferator-activated receptors
*PPARγ*	proliferator-activated receptor γ
*PPARγC1A*	PPARγ coactivator 1 alpha
PUFAs	polyunsaturated fatty acids
RNS	reactive nitrogen species
ROS	reactive oxygen species
*SCD*	stearoyl-CoA desaturase
SCFAs	short chain fatty acids
Se	selenium
SeMet	selenomethionine
SFAs	saturated fatty acids
*SLC27A6*	solute carrier family 27-member 6
*SOD*	superoxide dismutase
*SREBF1*	sterol regulatory element binding transcription factor 1
SS	sodium selenite
TAGs	triglycerides
*TALDO1*	transaldolase 1
TFs	transcription factors
*TNFA*	tumor necrosis factor alpha
*TXN*	thioredoxin
UFAs	unsaturated fatty acids
VLDL	very-low-density lipoprotein
*XDH*	xanthine dehydrogenase
